# Integrating Smart Worksheets into mandatory pre-laboratory exercises increased exercise completion rates and laboratory test grades

**DOI:** 10.1042/ETLS20253023

**Published:** 2026-02-26

**Authors:** Imaan Huseeb, Ines Ramos-Harrison, Andrew Kenny, Tanya Klymenko, Franziska Ludwig, Jef Clark, Emily Coyte, Leanne Williams, Rachel Schwartz-Narbonne

**Affiliations:** 1 Sheffield Hallam University, Sheffield, South Yorkshire, S1 1WB, U.K.; 2 LearnSci (Learning Science Ltd), Bristol, BS1 5DP, U.K.; 3 University of Warwick, Coventry, CV4 7AL, U.K.

**Keywords:** LearnSci, pedagogy, pre-laboratory, pre-labs, Smart Worksheets

## Abstract

Pre-laboratory exercises are assigned to promote student preparedness, but these provide no benefit if students do not engage. Students’ perceived barriers to pre-laboratory-exercise engagement were identified via analysis of open and closed survey questions; key issues were time burden and perceived complexity. To facilitate future engagement, students suggested making pre-laboratory exercises clearer and easier to access with stylistic changes, as well as making them mandatory.

Two interventions to first-year laboratories were implemented. First, LearnSci Smart Worksheets were deployed alongside traditional pre-laboratory exercises; these worksheets are interactive and scaffold learning. Second, the laboratory pass condition was changed from ‘70% physical attendance’ to ‘80% engagement’. Engagement points were awarded for physically attending laboratory sessions (3 points) and for completing pre-laboratory exercises (0.5 points for traditional exercise, 0.5 points for Smart Worksheet exercise). Passing thus required a combination of physical attendance and completion of pre-laboratory exercises.

Prior to the interventions, pre-laboratory exercises had low completion rates (16 ± 11% of students completed each monitored exercise). Post-interventions, 84 ± 3% of students completed each Smart Worksheet, and 79 ± 4% completed the traditional exercises. There was a statistically significant increase in mean laboratory test marks post-intervention (pre-intervention 65 ± 11%; post-intervention 72 ± 10%). Post-intervention, pre-laboratory exercise completion was significantly positively correlated with laboratory test marks. We hypothesise these two interventions increased incentives to engage with pre-laboratory exercises and so contributed to increasing student success.

## Introduction

Effective preparation for laboratory practical sessions is a critical factor influencing students' engagement, confidence and academic performance in science education [1]. These pre-laboratory exercises traditionally consist of textual instructions or procedural notes intended to familiarise students with laboratory protocols. However, these resources often fail to actively engage learners, leading to passive reading and limited retention of important practical information [2]. Active recall, actively retrieving information rather than passively reviewing it, has consistently been demonstrated as one of the most effective methods to enhance long-term memory retention [3]. Applying this approach to pre-laboratory preparation encourages students to engage deeply with the material, ensuring that critical laboratory concepts and techniques are not merely recognised but fully understood and remembered [4].

When students perceive pre-laboratory tasks as optional, it further reduces student engagement, limiting their effectiveness in promoting laboratory preparedness [2,5], as students may assign lower priority to non-grade bearing material. This is particularly acute when students face other challenges such as transition to university life [6], and when the material is unnecessarily complex and difficult to use [2,4]. Lack of engagement means students miss out on the benefits of identifying and addressing knowledge gaps, improving confidence and ultimately improving skills acquisition. It negatively affects the lab experience, as sessions need to allow students time to familiarise themselves with the materials and complete any required calculations, rather than completing these tasks during prep time.

**Figure 1:**
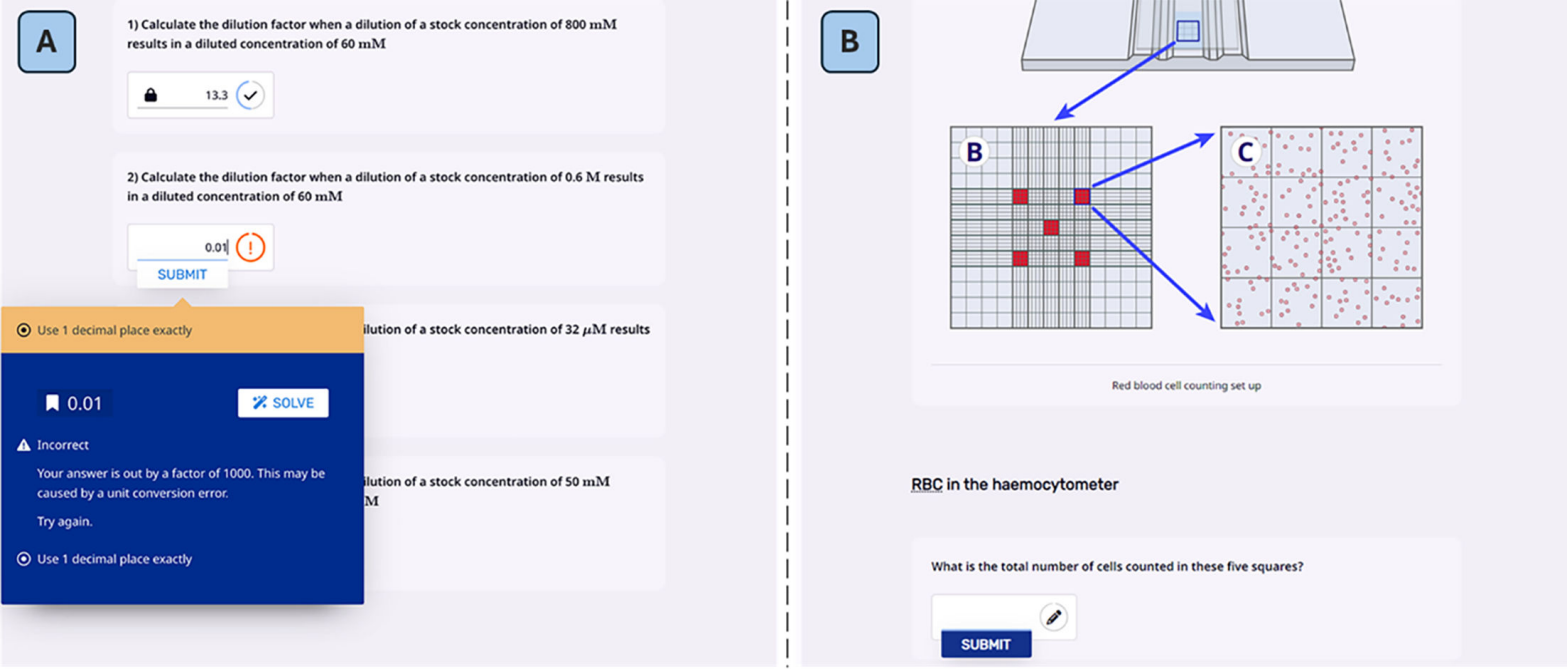
Example LearnSci Smart Worksheets on relevant topics including A) dilution factors and B) cell counting. Used with permission.

LearnSci’s (Learning Science Ltd.) Smart Worksheets are interactive worksheets that provide students with instant, personalised targeted feedback, as well as offering hints and guidance to support student learning (Figure 1) [7]. While academics can commission bespoke Smart Worksheets, access to the Smart Worksheet Library of resources can be more quickly utilised without the need for development and the associated staff time. This makes utilisation of the resource more accessible to academics who teach cohorts covering multiple courses with a diversity of laboratory sessions. Previous studies have utilised this technology to support post-laboratory data analysis [8–10] and to prepare students for classroom-based workshops [11,12]. When deployed as formative preparation materials, student grades were significantly higher for those individuals who engaged with the Smart Worksheets [10,11]. Their use also led to higher grades across cohorts [9,13].

## Educational context

Sheffield Hallam University School of Biosciences & Chemistry First Year cohort is composed of approximately 250 students from five courses: biomedical sciences (BMS; approximately 150 students per intake), three bioscience courses (Biology, Biochemistry, and Biomedicine & Health Science; 10–35 students per intake) and one Chemistry course (10–35 students per intake, per course). Level 4 Fall Semester includes between 15 and 17 laboratory sessions (15 sessions for BMS, 16 sessions for Biology, 16 sessions for Biochemistry, 15 sessions for Biomedicine & Health Science and 17 sessions for Chemistry), with each session consisting of a half or full day of laboratory work. Pre-laboratory exercises are provided and students are instructed to complete these before the laboratory sessions.

The laboratory sessions vary by course, with some overlap (e.g. all courses performed ‘Protein Determination’, only Chemists performed ‘Transition Metal Complex’ and all courses besides the Chemists performed ‘Bacterial Identification’). The BMS cohort was subdivided into five laboratory groups, and the order of the laboratory sessions varied slightly between groups. A visual representation is available in Supplementary Figure S1.

Previously, pre-laboratory exercises consisted of reading written materials including the laboratory manual. These materials were provided as physical books and electronically via the university’s virtual learning environment, Blackboard. Students were instructed to write notes on their reading. A subset of the pre-laboratory exercises included questions. Students were instructed to submit their answers on a Google Form embedded on Blackboard. The notes and form were not monitored.

Module assessment includes an end-of-module online open-book summative assessment, testing understanding of the laboratory sessions and the pre- and post-laboratory exercises.

In September 2024, Level 5 students were surveyed to provide a baseline understanding of students’ experiences of traditional pre-laboratory exercises. The interventions targeted Level 4 students during the 2024 Fall semester (September to December).

As technicians and lecturers responsible for laboratory teaching, we observed that, pre-interventions, students typically arrived at laboratory sessions unprepared: despite instructions, the concentrations of solutions they would need were not calculated, and students could not identify what techniques would be applied and the associated health and safety risks. This necessitated using laboratory time to perform calculations and familiarise students with the session, taking time away from hands-on skills acquisition. We also hypothesised it led to increased student anxiety and lowered student confidence as they started each session unsure of what they needed to do.

The aims of these interventions were to increase pre-laboratory exercise completion rates, with the hypothesis that this would increase student preparedness and therefore confidence and learning in the laboratory sessions. We hypothesised this would result in students achieving higher grades in the summative laboratory test.

As metrics, we measured pre-intervention and post-intervention pre-laboratory exercise competition rates and grades on the summative laboratory test.

## Interventions

**Figure 2:**
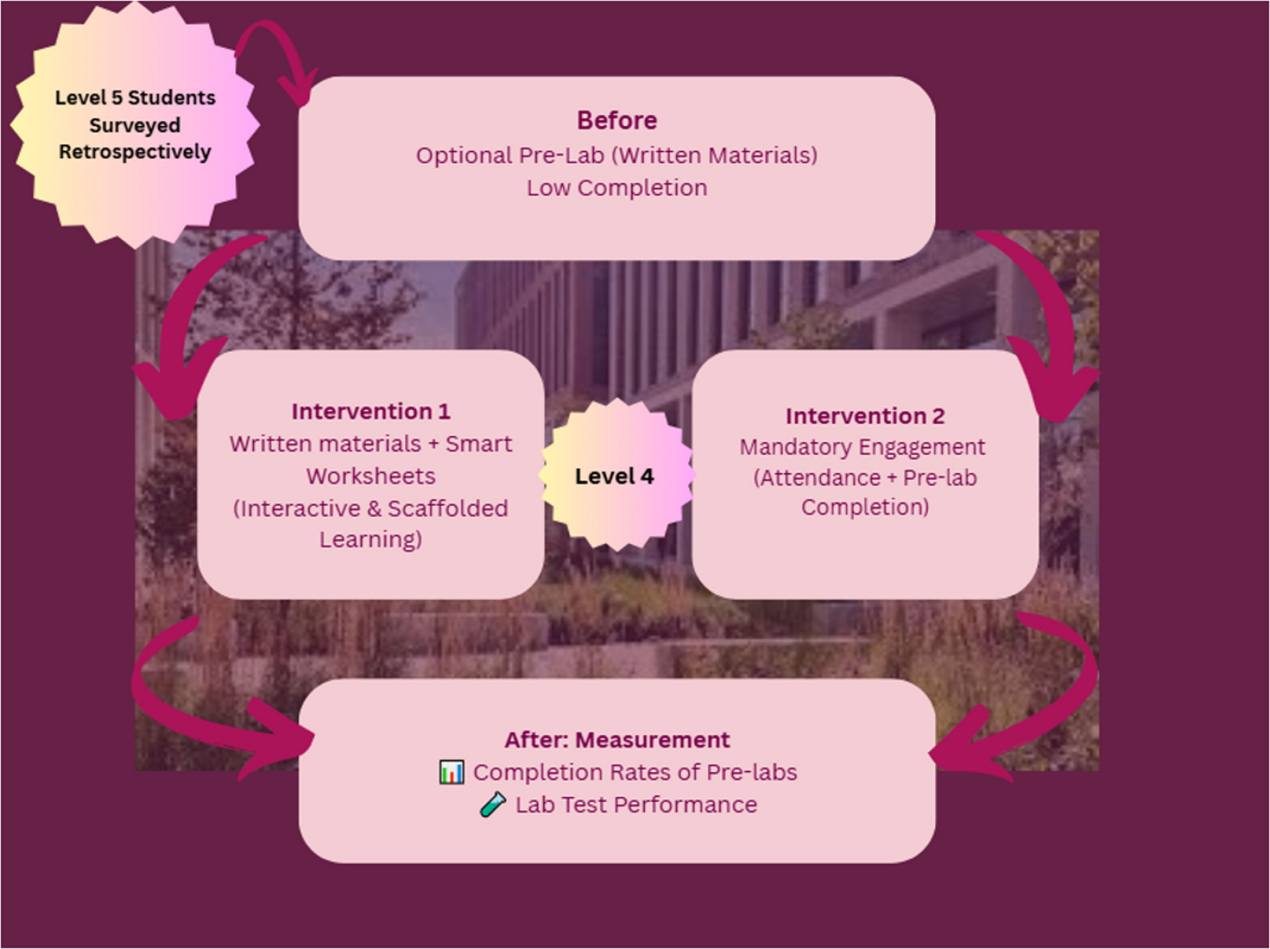
Sequence of measurements and interventions conducted within this study.

Two interventions were simultaneously deployed with the Level 4 cohort in the fall semester (Figure 2).

**Figure 3:**
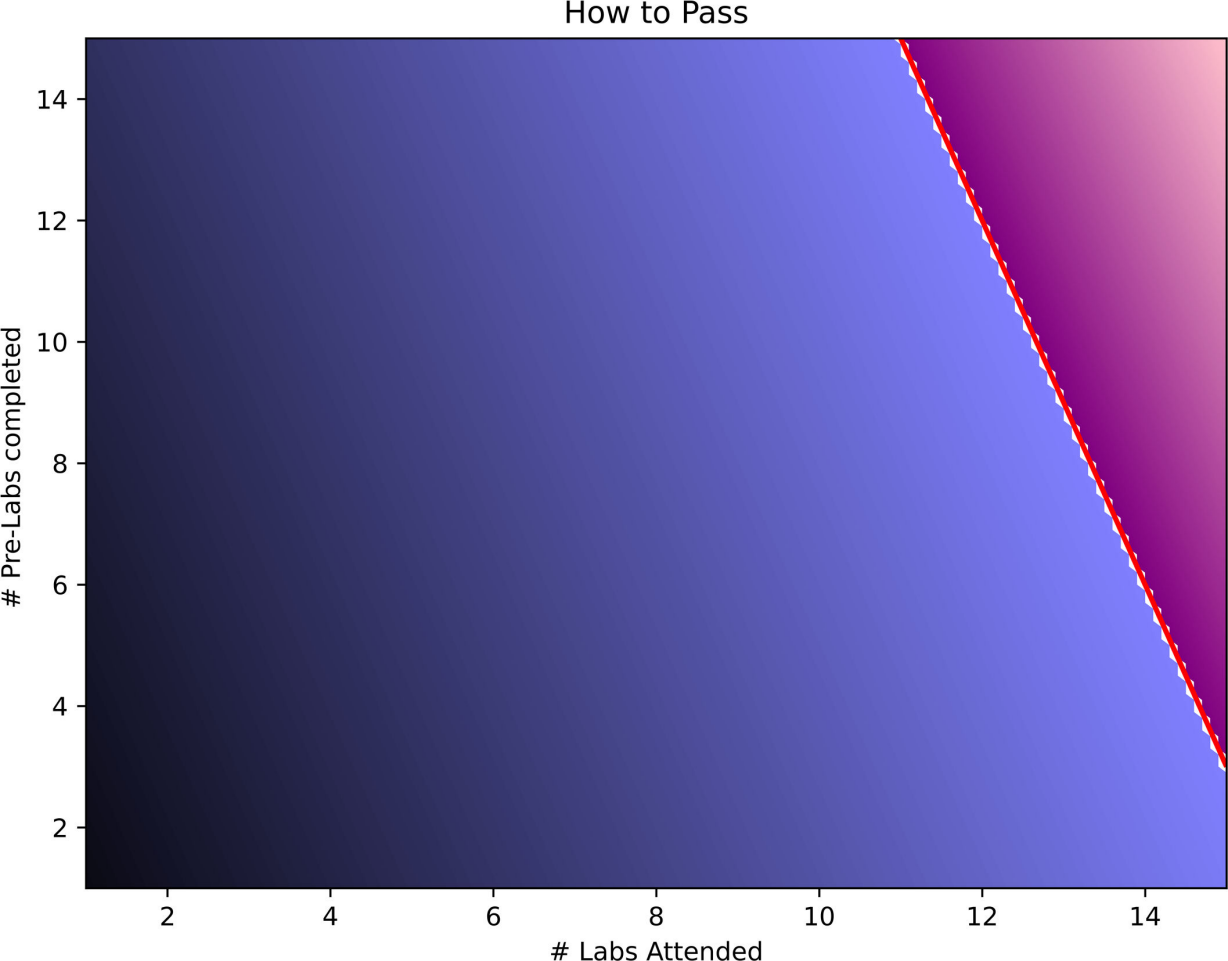
Combinations of physical laboratory attendance and pre-laboratory exercises that lead to a module pass (purple) versus fail (blue), assuming the course has 15 laboratory sessions. Note that traditional pre-laboratory exercises and Smart Worksheets each count as a 0.5 completion; there is no requirement to complete them both.

1. Each laboratory activity had one Smart Worksheet and one traditional pre-laboratory exercise associated with it. These were placed side by side in the same Blackboard folders. LearnSci Library Smart Worksheets were mapped to 26 distinct laboratory sessions to ensure alignment. Traditional pre-laboratory exercises were not changed besides introducing electronic submission of notes and answers on Blackboard. Completion of both exercise types was monitored via the grades section in Blackboard.
2. Pre-intervention, students were required to meet a 70% ‘laboratory attendance’ threshold to pass the module, managed by attendance register. This was changed to an 80% ‘laboratory engagement’ threshold, where students needed to achieve 80% of possible engagement points. For each laboratory session, 3 points were awarded for each half-day attending the laboratory, plus 0.5 points for completing the traditional pre-laboratory exercises and 0.5 points for completing the relevant Smart Worksheet (Figure 3).

## Methods

### Pre-intervention questionnaire deployment

Pre-intervention, student evaluation of pre-laboratory exercises was solicited via an anonymous Microsoft Forms questionnaire conducted with students at the start of Level 5. A mixture of closed and open questions was used to assess students’ perceptions of traditional pre-laboratory exercises and barriers to engagement with them.

### Data analysis

A final-year student researcher and study author, Imaan Huseeb, aided data analysis of student questionnaires, engagement and grades. The student researcher received full training and undertook the project as a part of her degree studies.

Four questionnaire questions (two open, two closed) were analysed. The open-ended survey responses were analysed using a summative content analysis approach [14] to quantify the occurrence of specific keywords and interpret their underlying meaning. This method was carried out by two coders, done by hand and using an Excel spreadsheet to capture results allowing for comparison and compilation of the data. First, the text data were systematically scanned to identify and code all relevant keywords based on their sentiment, classifying them as either positive or negative based on the context of the sentence. Following this initial quantification, these coded responses were then inductively grouped into seven broader thematic categories (e.g. ‘Utility’, ‘Confidence’, ‘Time’, ‘Difficulty’, ‘Interest’, ‘Clarity’ and ‘Feedback’) to move beyond simple word counts and capture the full scope of student feedback. The final analysis involved calculating the frequency of responses within each sentiment category and thematic group, allowing for a quantitative interpretation of the most prevalent positive and negative aspects of the student experience.

Engagement with pre-laboratory exercises and summative test grades was measured pre-intervention (2023–2024) and post-intervention (2024–2025). Pre-intervention, while students were expected to complete a pre-laboratory exercise for every laboratory session, the majority of these were completed within students’ paper laboratory books; only six pre-laboratory exercises had electronic submission points. As paper laboratory books were not checked, the overall completion rate pre-intervention could not be analysed. However, the anonymised completion data for the six electronically submitted pre-laboratory exercises was extracted and percentage completion was calculated. Post-intervention, all pre-laboratory exercises were submitted electronically on Blackboard. The pre-laboratory exercise engagement rate could only be compared between years for those six laboratory sessions. Post-intervention, it could be compared between type of pre-laboratory exercise for all laboratory sessions.

Student pre- and post-intervention performance on the summative test was downloaded from Blackboard and anonymised. Students who did not attempt the test were excluded from this analysis.

Statistical analysis and visualisation were performed using Python. A Shapiro–Wilks test indicated deviation from normality (SW = 0.976, *P* = 0.002) so non-parametric data analysis was performed: Wilcoxon signed-rank for non-parametric paired data, Mann–Whitney for non-parametric unpaired data. Wilcoxon signed-rank tests included a power calculation and reporting w-statistics, *P*-value and Cohen’s *d*. A non-parametric approximation of a power analysis was performed using an alpha of 0.05 and a power of 0.8.

Impact of interventions on laboratory test scores was determined using Mann–Whitney *U* test. The mean standard deviation, Rank Biserial correlation and *U* statistic were reported.

## Results

### Student perceptions of traditional pre-laboratory exercises

We aimed to improve engagement with and value of pre-laboratory exercises, anticipating that this would increase student learning during laboratory sessions and thus lead to an increase in skills, understanding and in associated grades.

The project began by surveying students’ perceptions of traditional pre-laboratory exercises and barriers to their engagement. Of the 197 students enrolled at the start of Level 5, 43 responded to at least one question (22% response rate). Answers were consolidated and analysed at the cohort level.

**Figure 4:**
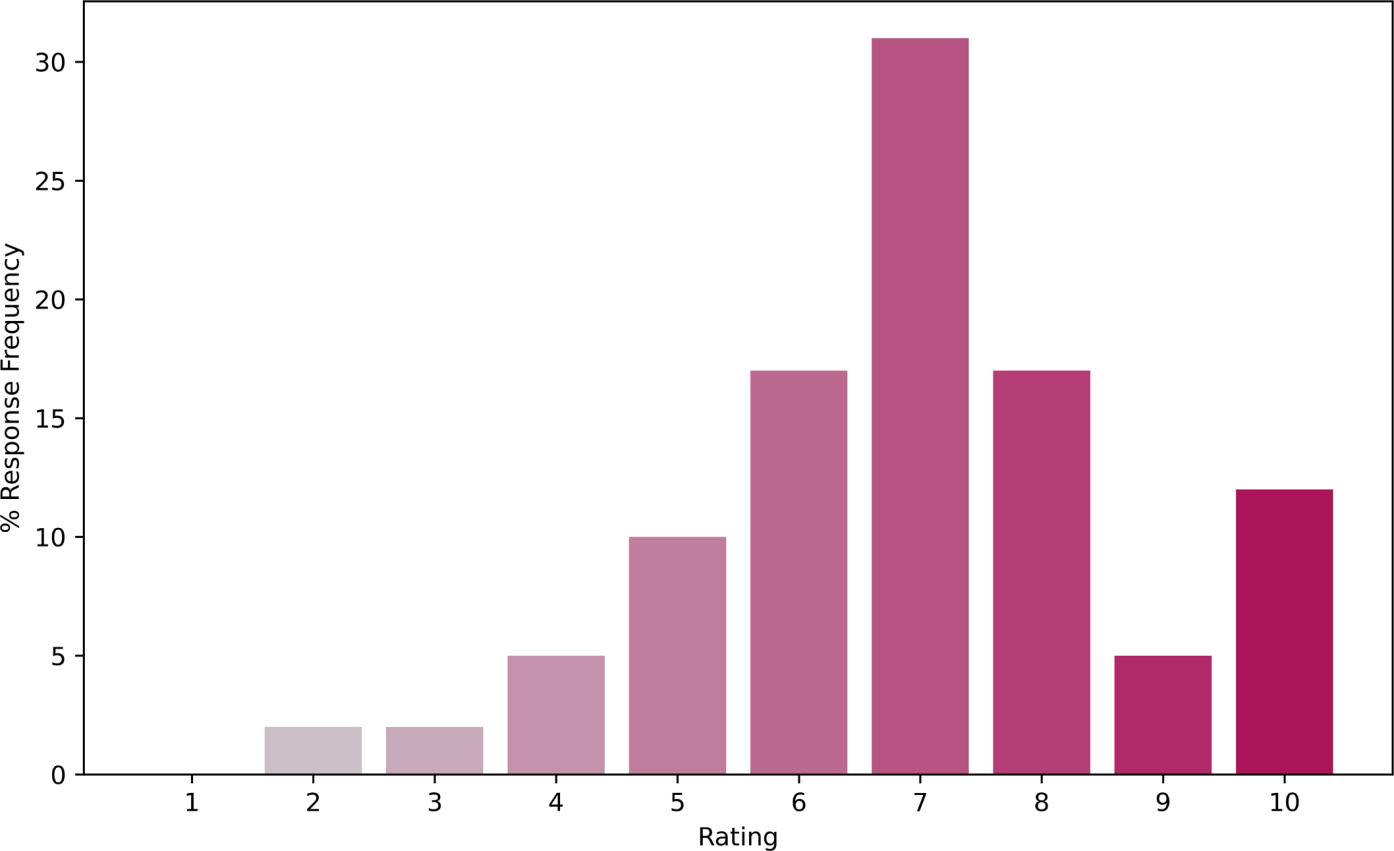
Pre-intervention student survey responses to the question ‘On a scale of 1 to 10, how much would you recommend these pre-laboratory resources to other students on a similar course?’ 1 was low and 10 was high (*N* = 42).

Students were asked to rate the traditional pre-laboratory exercises they had completed in Level 4 from 1 (low) to 10 (high) and explain their rating. The modal average rating was 7 (Figure 4). Qualitative content analysis of these responses demonstrates that a substantial proportion of students valued these resources, with the most common response mentioning their utility for the subsequent laboratory session (19 respondents, 73% of 26 respondents). However, students also highlighted negative aspects of the existing resources, with common responses including the difficulty level (15 %) and the lack of clarity in the resources (15 %), and some students finding the resources not useful (15 %). Multiple students also commented on the time-consuming nature of the work (12 %) (Table 1). The interventions in this study addressed some of these issues. LearnSci Smart Worksheets are designed for clarity and give feedback to scaffold students through difficulty. Mapping the Smart Worksheets onto the laboratory sessions meant they provided utility. However, no changes were made to the existing pre-laboratory exercises, and students were assigned both types of exercises. This increased the time required to complete the exercises, which did not align with student feedback.

**Table 1:** Content analysis of student responses to the open question ‘Please tell us why you gave the resources this [1 to 10] rating’ (*N* = 26)

Positive			Negative	
	Frequency: count (%)	Example	Frequency: count (%)	Example
Utility	19 (73 %)	It helped me to understand the lab task	4 (15 %)	They were not essential to completing the lab and gave little context around it.
Confidence	2 (8 %)	Help boost confidence …	1 (4 %)	... If the pre lab was particularly difficult it knocked my confidence...
Time	1 (4 %)	… not too long to do …	3 (12 %)	...They were time consuming often being multiple pages long…
Difficulty	0 (0 %)		4 (15 %)	I found them difficult so i didn’t really engage with them …
Interest	0 (0 %)		1 (4 %)	...there are some that are just bad or too tedious
Clarity	0 (0 %)		4 (15 %)	The pre labs were often confusing with little explanation provided…
Feedback	0 (0 %)		2 (8 %)	... we weren’t always given feedback on them

Students were asked a closed question regarding their perceived barriers to pre-laboratory exercise engagement. The most common response was ‘I didn’t have time’ (15 respondents, 45% of 33 respondents), followed by ‘They looked too difficult’ (30 %) (Table 2).

**Table 2:** Frequency analysis of student responses to the closed question ‘Please tell us why you didn’t use the pre-lab resources. Check all that apply’ (*N* = 33)

	Frequency: count (%)
I didn’t have time	15 (45 %)
They looked too difficult	10 (30 %)
I struggled to find them	8 (24 %)
They were not mandatory	8 (24 %)
They didn’t look interesting	7 (21 %)
They didn’t seem valuable to my learning	7 (21 %)
They didn’t work properly	5 (15 %)
They looked too easy	3 (9 %)
I didn’t know about them	2 (6 %)
This question does not apply to me	1 (3 %)
Did pre-lab	1 (3 %)

Student suggestions to facilitate higher engagement with pre-laboratory exercises were solicited (Table 3). Suggestions highlighted changes that would increase student ease of use, including accessing the resources, increasing the clarity of resources, and extra support. They also suggested increasing the interest level of the resources and increasing the utility of the resources, both in the materials themselves and in how feedback was provided. Adding LearnSci Smart Worksheets to the pre-laboratory exercises addressed some of these suggestions, as they are more interactive than traditional pre-laboratory exercises and provide feedback and tips to scaffold students. However, the existing resources were not improved at this point, as it was out of scope for the intervention. Additionally, students suggested making pre-laboratory exercises mandatory; this suggestion was followed.

**Table 3:** Content analysis of student answers to the open question, ‘What can we do to facilitate your engagement with the pre-lab resources?’ (*N* = 20)

	Frequency: count (%)	Example
Make them clearer	6 (30 %)	word the questions a bit clearer
Change the style	5 (25 %)	Potential games/interactive resources
Make them mandatory	4 (20 %)	make them mandatory
Facilitate access	4 (20 %)	Make them pop up prior to the lab rather than having to search for them
Increase utility	3 (15 %)	Make them more useful for the lab
Change delivery of feedback	2 (10 %)	Have the answers provided after the lab to correct ourselves if we got prelab questions wrong.
Provide more support	2 (10 %)	work through with others

### Impact of interventions on pre-laboratory completion

**Figure 5:**
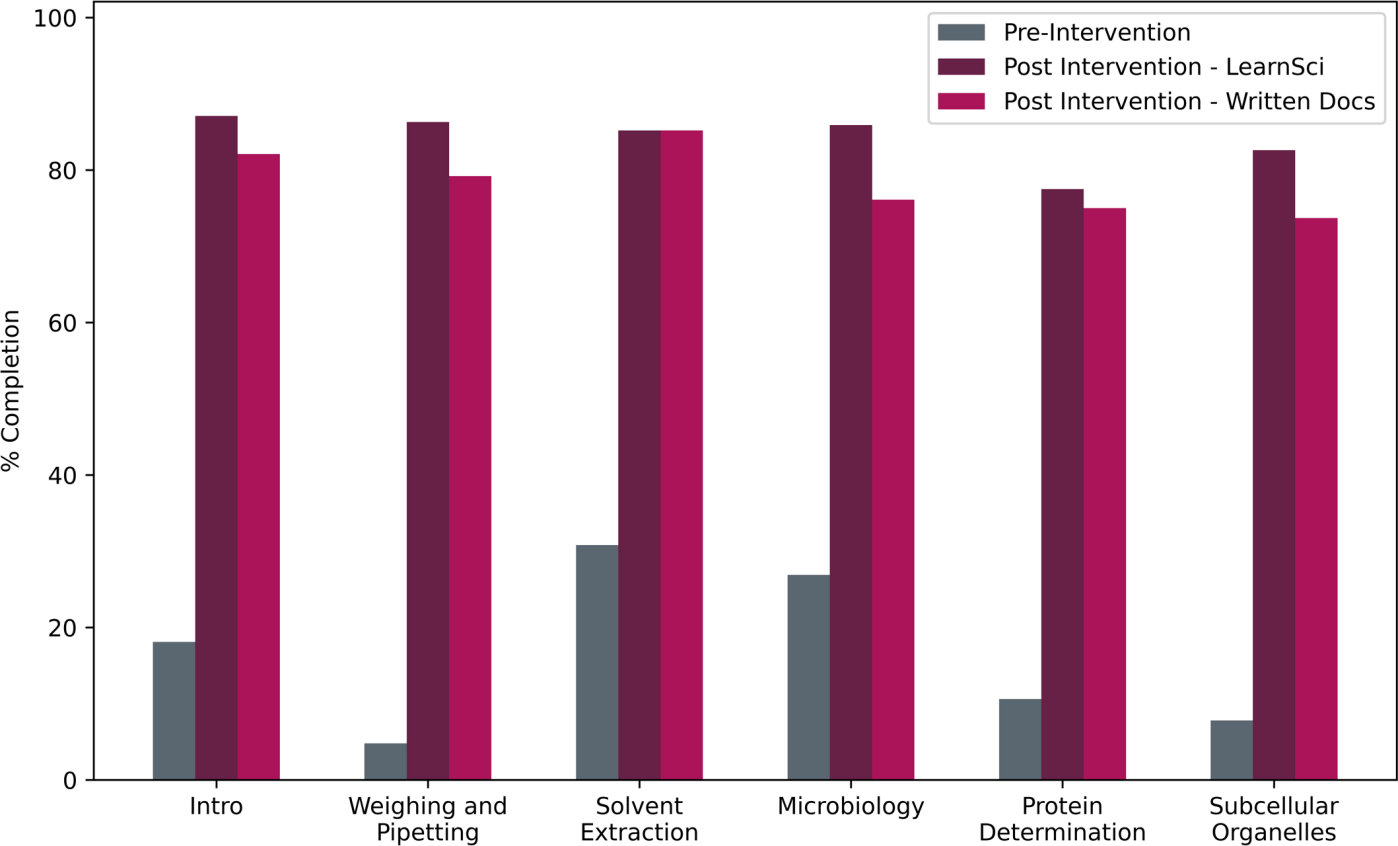
Comparison of pre-laboratory exercise completion rates for 6 laboratory sessions. A significant increase (*P*<0.05) in completion rates is seen from pre-intervention traditional exercises to post-intervention for both types of pre-laboratory exercises (Wilcoxon signed-rank test; w-statistic 0, power calculation = 1). Number of students assigned each pre-laboratory exercise varied between years and sessions. Intro, Weighing & Pipetting, and Protein Determination were completed by all courses (pre-intervention 271 students; post-intervention 240 students). Solvent Extraction (Chemistry) was completed by Chemists (pre-intervention 26 students; post-intervention 27 students). Microbiology and Subcellular Organelles were completed by Biomedical Scientists, Biologists, Biochemists, and Biomedical & Health Scientists (pre-intervention 245 students; post-intervention 213 students).

A subset of six exercises (where both pre- and post- intervention data were available) was compared to assess completion rates between years and types of exercise (Figure 5).

Pre-intervention, completion rates were low (17 ± 11% of students completed each monitored exercise). Post-intervention, there was a significant (Wilcoxon signed-rank test, *P* = 0.03) increase in completion rates, as 84 ± 4% of students completed each Smart Worksheet, and 79 ± 4% completed the traditional laboratory exercises. Applying the same paired Wilcoxon signed-rank testing to the comparison between LearnSci Smart Worksheets and the written documents did not return a significant difference (w-statistic 0, *P*=0.06, power calculation = 0) which, given the small sample size and marginal difference between the datasets, cannot be interpreted with confidence.

### Impact of interventions on grades

**Figure 6:**
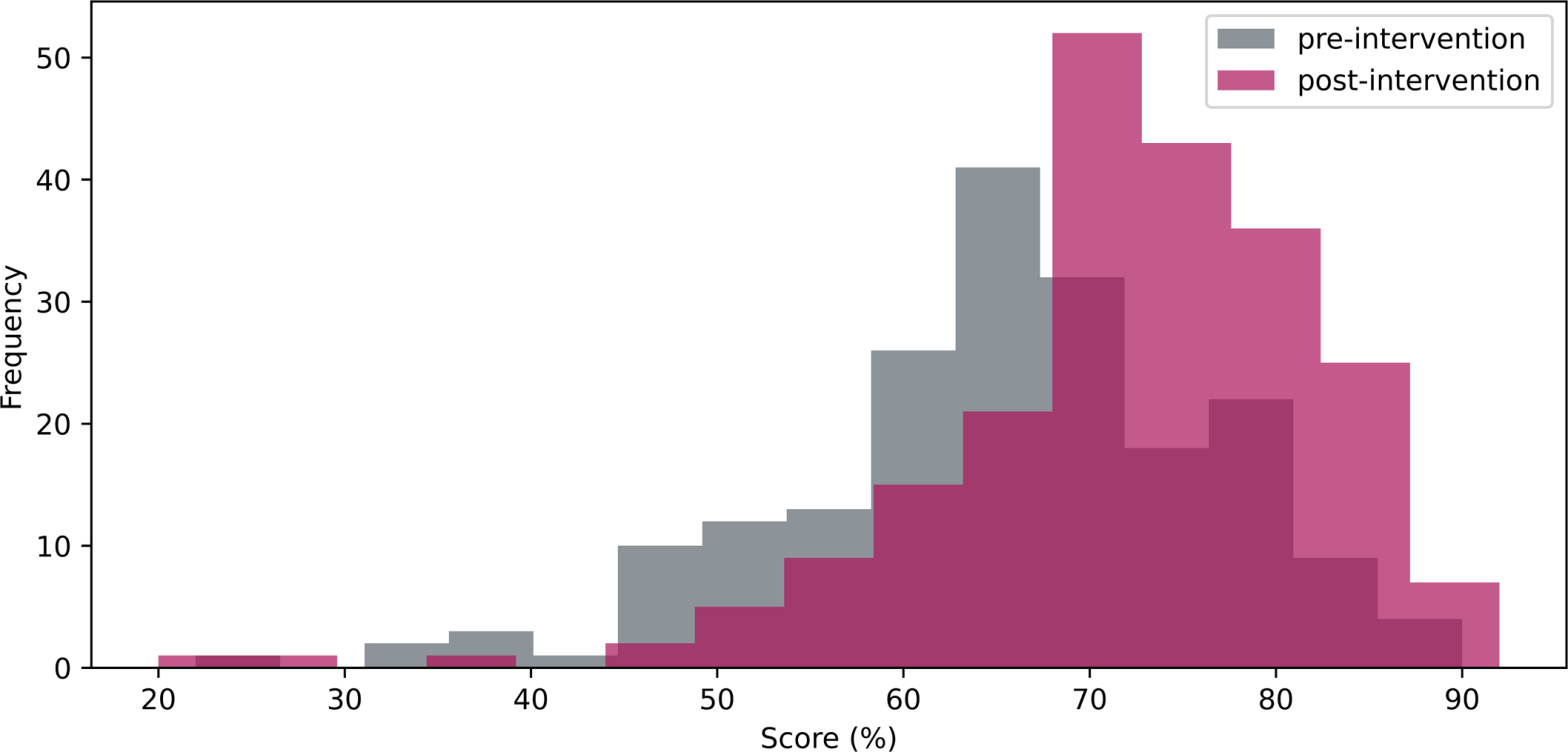
Pre- and post-intervention laboratory test scores. A significant increase (*P*-value < 0.05) was observed in average grade post-intervention.

Pre- and post-intervention scores on the open-book summative test were compared (Figure 6); using a Mann–Whitney *U* test resulted in a Rank Biserial correlation of 0.372 (*U* statistic 13,274 from 42,292). A significant increase (*P*-value < 0.05) was found post-intervention (pre-intervention 65 ± 11%; post-intervention 72 ± 10 %). The mapping of both styles of pre-laboratory exercises to the summative test is presented in Supplementary Table S1.

**Figure 7:**
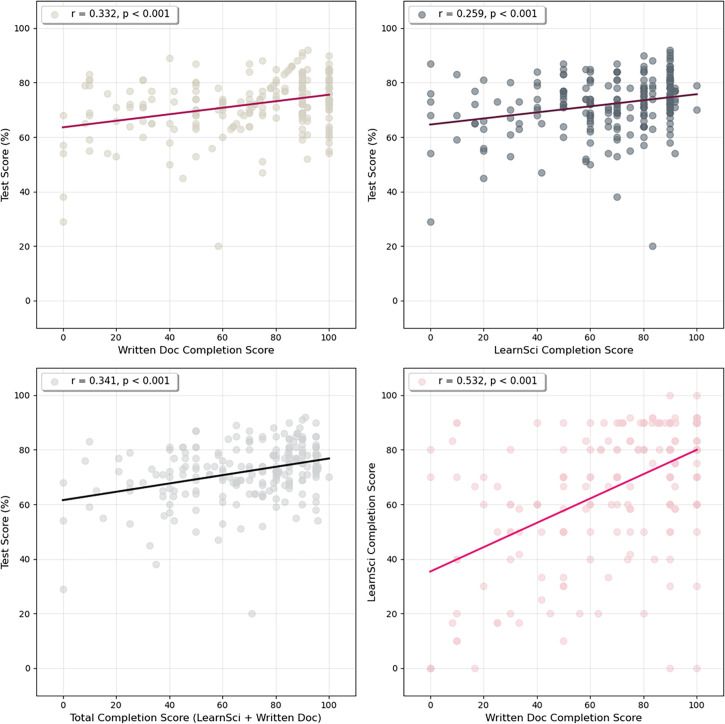
Correlation between percentage pre-laboratory exercise completion and summative test score in the post-intervention cohort (218 students) for (top left): traditional written pre-laboratory exercises, (top right): LearnSci Smart Worksheets (all pre-laboratory exercises (bottom left). Note that the percentage completion was scaled to 100% for each exercise type. The bottom left graph shows the correlation between completion of written pre-laboratory exercises and LearnSci Smart Worksheets.

The post-intervention impact of pre-laboratory exercise completion on summative test scores was calculated by correlating the percentage completion with a student’s final grade (Figure 7). Students who did not take the summative lab test during the first sit were excluded from the analysis. There was a small but significant positive correlation between pre-laboratory exercise completion and test score (Pearson correlation coefficient *r* = 0.341; *P* = 2.42e-7) for the cohort (218 students).

Interestingly, comparing the correlation between the completion of the LearnSci Smart Worksheets (*r* = 0.259, *P* = 1.082e-4) with the traditional pre-laboratory exercises for the whole cohort revealed a slight difference in the correlation (*r* = 0.332, *P* = 5.189e-7). Steiger’s test for dependent correlations revealed that the association between the traditional pre-laboratory exercise completion and test scores was significantly stronger than between Smart Worksheets and test scores, although the difference in impact on test scores was modest (Δr = 0.073); *z* = 0.715, *P*<0.001.

Comparing the completion of traditional written documents to LearnSci Smart Worksheets (Figure 7) shows a Pearson's correlation of 0r = .532, with 17.9% agreements at the ±5% level, 49.5% agreement at ±10% and 50.5% at ±15%. There is a calculated bias of 3.87% indicating a slight preference for the traditional written exercises.

### Discussion

Students’ perceptions of the pre-intervention pre-laboratory exercises aligned with findings of previous pedagogical studies; students perceived their utility [1] but critiqued them as unengaging and optional (Tables 1 and 2) [2,5]. Students also highlighted the lack of timely and consistent feedback on their answers (Table 1).

Post-intervention saw a dramatic increase in student pre-laboratory-exercise completion rates (Figure 5). Requiring students to complete some portion of these exercises to pass the module raised the intrinsic and extrinsic value students placed upon them, thereby fostering accountability and sustained participation [15], in alignment with literature that students respond positively and significantly to explicitly mandated tasks [2,5]. The digital resources may have appealed to students, as they addressed several of their issues regarding the traditional pre-laboratory exercises (Tables 1 and 3). Despite students describing time limitation as a major barrier to engagement (Table 2), increasing the quantity of pre-laboratory exercises to include both traditional exercises and Smart Worksheets did not counteract the other benefits of the intervention on competition rates.

Anecdotally, the study authors observed benefits from the increased student preparedness. Students came to labs with calculations tables filled in, saving approximately twenty minutes of lab time. Student confidence and competence were higher in performing follow-up calculations during the laboratory session. Demonstration time was also saved as students arrived aware of the general overview of techniques to be used. A follow-up study using focus groups to assess the impact of the intervention on both educators and students would allow these anecdotes to be more fully explored.

This study aligns with recent research highlighting structured preparatory tasks' role in reducing laboratory anxiety, enhancing skill acquisition and promoting deeper learning [16]. Effective pre-laboratory materials are increasingly recognised as essential for enhancing student preparedness, confidence and overall academic performance in science education. Recent studies consistently highlight the educational advantages of structured, interactive and mandatory preparatory exercises [1,5,16], consistent with the benefits observed in this study. While this study only demonstrates correlation between increased pre-lab engagement (Figure 5) and improved test outcomes (Figures 6 and 7), not causation, the experience of the teaching team suggests the intervention benefited students. While traditional written resources show a slightly stronger correlation with performance than Smart Worksheets alone – likely due to their direct alignment with lab tests – the LearnSci Worksheets still demonstrate robust correlation with student success. This suggests that the digital worksheets successfully facilitate the skills acquisition required for the curriculum without the requirement to commission bespoke materials. The data conclude, however, that a blended approach using both traditional and digital resources yields the best results.

## Study limitations

As both interventions were implemented simultaneously, it was impossible to fully distinguish the relative impact of the mandatory nature of the pre-laboratory exercises *versus* the addition of Smart Worksheets as part of the exercises. If, in future, other institutions implement only one aspect of this intervention, it would be worthwhile for them to analyse its impact.

Only one year of data are included in this study, and there may be cohort-level differences resulting in the higher grades for the summative laboratory test. In particular, the post-intervention cohort (2024–2025) is one year further away from the impacts of COVID-19 lockdowns on student learning. Given the limited scale of the study (covering only one pre-intervention and one post-intervention cohort), this data cannot distinguish such cohort-level effects.

## Conclusion

Pre-laboratory exercises are often assigned to support students with confidence and learning in laboratory sessions but provide no benefit if students do not engage. We asked students who had experienced optional, traditional pre-laboratory exercises what could increase their engagement. Common responses included changing the difficulty and format of the exercises, as well as making them mandatory (Table 3).

An intervention was deployed targeting these areas. Pre-laboratory exercise completion became a portion of the overall ‘laboratory engagement’ required to pass the module and traditional pre-laboratory exercises were supplemented with LearnSci Smart Worksheets.

The pre-laboratory exercises being made mandatory are likely responsible for their increased completion rates, as 17 ± 11% of students completed traditional exercises when they were optional, and 79 ± 4% when they were mandatory (Figure 3). The post-intervention slightly higher completion rate for Smart Worksheets than traditional Word documents (84 ± 4 %) was not statistically significant (>0.5). Importantly, despite the increased workload for students, engagement increased for both types of exercises.

This intervention correlated to increased average grades in an online laboratory test. The number of fail grades (>40 %) decreased from 3 % to 1 %, supporting the intervention having a positive impact on retention and progression.

## Supplementary material

online supplementary material 1.

## Data Availability

Anonymised data will be made available upon request.

## Open Access

This article has been published open access under our Subscribe to Open programme, made possible through the support of our subscribing institutions, learn more here: https://portlandpress.com/pages/open_access_options_and_prices#conditional

## Abbreviations

BMS: biomedical sciences
