# Supplementary material for: Integrating Smart Worksheets into mandatory pre-laboratory exercises increased exercise completion rates and laboratory test grades
Source: Emerg Top Life Sci. 2026 Feb 26;9(5):ETLS20253023. doi: 10.1042/ETLS20253023 (PMC13043652; doi:10.1042/ETLS20253023)
Supplement: online supplementary material 1. [file ETLS-9-5-ETLS20253023-s001.docx]

## Integrating Smart Worksheets into mandatory pre-laboratory exercises increased exercise completion rates and laboratory test grades

## Supplementary Information


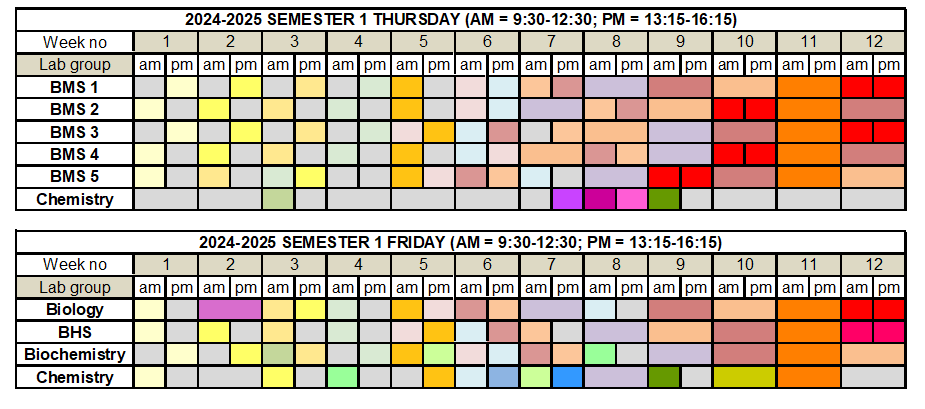


Supplementary Figure 1. 2024-2025 semester 1 laboratory sessions, divided by lab group and course. Pale grey represents no session; all other colours represent distinct laboratory sessions. Note that the summative test covered material from Week 1 to Week 9.

Supplementary Table 1. 2024-2025 semester one mapping of pre-laboratory exercises to laboratory sessions, and mapping of each type of pre-laboratory exercise to the summative test questions. Note that which labs were undertaken varied by course, and the order varied by lab group (see visual representation in SI Fig. 1). In addition to the exercises described, students were always assigned the task of reading and summarizing the lab script as part of their written documentation. The summative laboratory test also covered post-laboratory exercises, but analyses of these are out of the scope of this study.

| **Laboratory Session** | **LearnSci Smart Worksheet** | **Link to Summative Test** | **Written Docs** | **Link to Summative Test** |
| --- | --- | --- | --- | --- |
| Anatomy | Averages, Spread and Precision (Biomed) | Questions on accuracy & precision | Summarize microscopy information & biological drawing guidance | N/A |
| Bacterial Identification | Histological staining | N/A | Identify aims & risks; Questions on bacterial identification techniques | N/A |
| Conductivity | Determine an Unknown Concentration of Acid by Titration | N/A | Identify aims & risks; Questions on theory; Calculations | Questions on theory |
| EDTA | Acid Base Titration | N/A | Identify aims & risks; Questions on theory; Calculations | N/A |
| Electrochemistry | Redox and electrochemistry | Questions on electrochemical cells | Questions on electrochemistry | Questions on electrochemistry |
| Formative Test | N/A | N/A | N/A | N/A |
| Intro - Microbiology | Serial Dilutions | Dilution calculations | Summarize aseptic techniques | N/A |
| Intro - Microscopy | Microscopy: Estimating Size | N/A | Summarize microscope setup and usage | N/A |
| Intro - Reflux and Recrystallisation | Synthesis of Paracetamol | Questions on disposal routes; IR spectroscopy analysis | Identify aims & risks | N/A |
| Intro 1 | Moles and Molarity | Molar calculations | Summarize pipetting information | Read pipette volumes |
| Intro Weighing & Pipetting | Single Step Dilutions | Dilution calculations | Summarize solution preparation techniques | Molar and dilution calculations |
| Irn Bru | Beer-Lambert equation | N/A | Identify aims & risks; Describe theory & predict colours changes | N/A |
| Oil of wintergreen | Infrared Spectroscopy: Analysis | IR spectroscopy analysis | Identify aims & risks; Describe and explain key reaction & purification steps | N/A |
| Peak District Field Trip | Simpson's biodiversity index | N/A | N/A | N/A |
| pH & Buffers | Determine an Unknown Concentration of Acid by Titration | N/A | Identify aims & risks; Describe theory | N/A |
| Protein Determination | Variability and Confidence Intervals | N/A | Identify aims & risks; Describe theory; Practice calculations | N/A |
| Solvent extraction (Biochemistry) | Units and Unit Conversions (Chemistry) | Unit conversion calculations | Identify aims & risks; Describe and explain key reaction & purification steps | N/A |
| Solvent extraction (Chemistry) | Averages, Spread and Precision (Chemistry) | Questions on accuracy & precision | Identify aims & risks | N/A |
| Subcellular Organelles | Dilution factors | Dilution calculations | Calculations; Techniques flow chart; summarize centrifuge technique | Questions on techniques; Questions on calculations |
| Thermodynamics | Data Visualisation and Graphing (Chemistry) | N/A | Describe theory; Practice calculations | N/A |
| Transition Metal Complex | Coordination compounds | N/A | N/A | N/A |
| Clinical Biochemistry - Part A | Mass spec fundamentals | Delivered after test | Apply preliminary data to diagnose patients; Calculations | Delivered after test |
| Clinical Biochemistry - Part B | N/A | Delivered after test | Summarize HPLC information; Apply preliminary data to diagnose patients; Calculations | Delivered after test |
| Column Chromatography & Optical Isomerisms | Naming multi-chain molecules | Delivered after test | Questions on purification techniques & organic chemistry | Delivered after test |
| Human Physiology | Scientific Numeracy | Delivered after test | Summarize effects of exercise on human systems; complete physical readiness questionnaire | Delivered after test |
| Investigative Biochemistry - Part A | Mass spec fundementals | Delivered after test | Apply preliminary data to diagnose patients; Calculations | Delivered after test |
| Investigative Biochemistry - Part B | N/A | Delivered after test | Summarize HPLC information; Apply preliminary data to diagnose patients; Calculations | Delivered after test |
| KIT assignment | NA | Delivered after test | Ethics; Risk assessment; Costings; Sustainability | Delivered after test |
| Lac operon | Data Visualisation and Graphing (Biomed) | Delivered after test | Describe theory & predict colour changes | Delivered after test |
| Synthesis of Esters | Infrared Spectroscopy: Analysis | Delivered after test | Identify aims & risks; Describe theory | Delivered after test |

## References
